# On Indigestion, and Certain Bilious Disorders Conjoined with It, &c.

**Published:** 1847-04-01

**Authors:** 


On Indigestion and certain Bilious Disorders conjoined with it; to which are added Short Notes on Diet. By *G. C. Child,* M.D. Physiciam to the Westminster Dispensary. Octavo, pp. 219. London: Churchill, 1847.

The author has evidently expended much care and labour in the preparation of his work. Although not containing anything with which every medical man of any experience is not perfectly conversant, its contents certainly evince, on the whole, an attentive observation of the phenomena of Dyspepsia, and a sound judgment in their discrimination and treatment. Dr. Child attempts, on several occasions, to reduce the study of the symptoms of this protean disease to a standard of greater accuracy and exactitude than has been done before, by frequent reference to the statistical data furnished by 200 cases, of which he has kept a minute register. It is, however, very questionable whether any good can ever result from such elaborate divisions and sub-divisions as are found in the following passage on the subject of the "various pains observed in indigestion:"

"All the terms may be arranged in six groups.

I. Weight about the pit of the stomach, or front of the chest—and as more or less synonymous with this, may be regarded,
 Distention.
 Tightness.
 Oppression.
 Pressure.
 Stoppage; the lodging of food, as if it would pass no further than the epigastrium.

II. Aching.—Gnawing.
 A dull, wearing, or 'dead' pain.
 A dragging or 'heavy' pain.

III. Sensation of heat (*fer chaud* of French writers).
 Burning or hot pain.
 Acridity.
 Rawness, &c.

IV. A sensation like cramp.
 Spasms (often applied by patients to sharp pain).
 As if the stomach were fixed or nailed to, or drawn up against, the spine; as if it were turned into bone; as if a bone were in it.
 A ball, bullet, or something hard at the epigastrium, or under the sternum.
 A knot in the same place.
 A drawing pain, forcing the patient to stoop, or 'bending him double.'
 A severe dragging, twisting, or tearing pain.
 As if a cord were drawn tightly round the waist.

V. Sharp pains.
 Spasms (used also to denote cramp).
 Acute, quick, or darting pain.
 Plunging, twinging, or pinching.
 Like pins and needles (often an anomalous pain).

VI. Anomalous pains form a less numerous class than one might expect, and are chiefly observed in the nervous, hysterical, and hypochondriacal. They are insufficient of themselves to characterise dyspepsia, and hence are always associated with some of the other pains above-mentioned.
 Beating, fluttering, hammering, pulsation.
 'A moving' in the stomach: 'nerves working.'
 Tumbling, trickling.
 A sensation of cold at the pit of the stomach.
 A sinking, emptiness, or numbness at the same place.

"Table showing the comparative frequency of the different kinds of pain above-mentioned in 200 well-marked cases of indigestion:—

| | |
|---|---|
| Weight or oppression in .. .. .. | 160 cases. |
| Gnawing or aching .. .. .. .. .. | 121 " |
| Sharp pains .. .. .. .. .. .. .. | 95 " |
| Hot or acrid sensations.. .. .. .. | 40 " |
| Crams or spasms .. .. .. .. .. | 26 " " |

Such labour is almost like that of one seeking to describe the thousand forms of clouds in a summer sky, or the ever-varied hues upon a pigeon's breast. The "Short Notes on Diet," were surely not intended for the instruction of medical man; if designed for other readers, they should not have found a place in a work addressed to the profession.

